# Development of In Vitro Assays for Advancing Radioimmunotherapy against Brain Tumors

**DOI:** 10.3390/biomedicines10081796

**Published:** 2022-07-26

**Authors:** Yohan Walter, Anne Hubbard, Allie Benoit, Erika Jank, Olivia Salas, Destiny Jordan, Andrew Ekpenyong

**Affiliations:** 1 Department of Physics, Creighton University, Omaha, NE 68178, USA; yohanwalter@creighton.edu (Y.W.); annehubbard1@creighton.edu (A.H.); alliebenoit@creighton.edu (A.B.); erikajank@creighton.edu (E.J.); oliviasalas@creighton.edu (O.S.); 2 Department of Biology, Creighton University, Omaha, NE 68178, USA; destinyjordan@creighton.edu

**Keywords:** glioblastoma, immunotherapy, radiotherapy, brain cancers, radioimmunotherapy, immune checkpoint inhibitors, temozolomide, durvalumab, immunoradiotherapy

## Abstract

Glioblastoma (GBM) is the most common primary brain tumor. Due to high resistance to treatment, local invasion, and a high risk of recurrence, GBM patient prognoses are often dismal, with median survival around 15 months. The current standard of care is threefold: surgery, radiation therapy, and chemotherapy with temozolomide (TMZ). However, patient survival has only marginally improved. Radioimmunotherapy (RIT) is a fourth modality under clinical trials and aims at combining immunotherapeutic agents with radiotherapy. Here, we develop in vitro assays for the rapid evaluation of RIT strategies. Using a standard cell irradiator and an Electric Cell Impedance Sensor, we quantify cell migration following the combination of radiotherapy and chemotherapy with TMZ and RIT with durvalumab, a PD-L1 immune checkpoint inhibitor. We measure cell survival using a cloud-based clonogenic assay. Irradiated T98G and U87 GBM cells migrate significantly (*p* < 0.05) more than untreated cells in the first 20–40 h post-treatment. Addition of TMZ increases migration rates for T98G at 20 Gy (*p* < 0.01). Neither TMZ nor durvalumab significantly change cell survival in 21 days post-treatment. Interestingly, durvalumab abolishes the enhanced migration effect, indicating possible potency against local invasion. These results provide parameters for the rapid supplementary evaluation of RIT against brain tumors.

## 1. Introduction

Cancer is the second leading cause of death in the United States, presenting a major concern both on the individual scale and for public health [1]. The National Cancer Institute estimated over 1.8 million total new cases, with over 600,000 deaths due to cancer in the US alone, for the year 2020 [1]. Despite modern advances in diagnostic technology and cancer therapy, there are still many cancers for which therapy is not effective, such as glioblastoma, a highly malignant primary brain cancer [2,3,4,5]. Cancer is a remarkably diverse disease, presenting over 200 unique types [1,6], each with differences in physical and biological characteristics. Moreover, cancer is, by nature, highly susceptible to mutation, which further broadens the spectrum of cases. Variations have even been found in individual response to treatment when comparing cancer from the same tissues of origin [6,7]. For example, temozolomide (TMZ), a well-known anticancer agent for glioblastoma, has been shown to be effective only in approximately 55% of patients [7]. Other mutations, including those which result in a gain of motility, are of significant prognostic concern, potentially increasing lethality, while affecting the efficacy of many treatment techniques. Gain of motility is implicated in lethality via metastasis.

Metastasis is the complex process by which cancerous cells migrate from primary tumors, spreading to form new tumors in non-contiguous, distant sites [8]. It is regarded as the primary factor implicated in cancer mortality, contributing to approximately 90% of cancer-related deaths [8,9,10]. Additionally, advanced metastatic cancer is rarely curable, despite modern advances in medicine, and most patients, at the time of diagnosis, present with metastatic cancer [8]. Major steps in the metastatic process include invasion, intravasation, extravasation, and colonization [11], all of which involve cell migration. Enhanced cell migration is a hallmark of malignant metastatic tumors including glioblastoma (GBM). In fact, GBM is a highly malignant primary brain tumor that is notoriously radioresistant and chemoresistant [3,12,13,14]. Combined with its resistance to treatment, glioblastoma’s lethality and invasiveness have made curative therapy impossible with current methods [2,3,4]. To date, there have been no cured glioblastoma patients, indicating a failure of the current treatment modalities and a radical need for improved therapeutic strategies against GBM and similar brain tumors.

Currently, the three main treatment modalities for cancer are surgery, chemotherapy, and radiation therapy. Since its inception in medicine, and recent developments including efforts resulting in a 2018 Nobel Prize [15], immunotherapy has arisen as a potential fourth modality [16]. Immunotherapy presents a versatile, potent mode of therapy which offers systemic coverage of the body [16,17]. Immunotherapy is emerging as a major breakthrough in therapy, contributing to modern reductions in cancer mortality, chiefly as a result of the successful treatment of metastatic melanoma using immune checkpoint inhibitors [1]. Additionally, combinations of immunotherapeutic strategies with the other three modalities, especially radiation therapy, have been fruitful in treating cases such as metastatic melanoma and non-small-cell lung cancer [18,19,20]. Unsurprisingly, owing to its lethality and resistance to treatment, GBM has become a target of interest for combination therapies including radioimmunotherapy (RIT), the combination of radiation therapy and immunotherapy, for treating disease [2,7,20,21,22,23,24,25]. Currently, phase I and phase II clinical trials using immune checkpoint inhibitors in RIT for GBM are ongoing in the United States, but many of the agents under these trials have simply been adopted owing to their safety and efficacy in treating other cancers [2,2]. Cellular-level radiobiological effects of RIT on GBM cells have not been fully explored. In order to provide assays for the rapid preclinical evaluation of RIT strategies, we hypothesized that the cellular-level effects of immunotherapeutic agents alone or in combination with chemotherapy and radiotherapy can provide readouts indicative of therapeutic potential.

To investigate this hypothesis, we monitored cellular-level effects in two well-characterized GBM cell lines, U87 MG [26,27] and T98G [28,29], following treatments with sublethal (5 Gy) and lethal (20 Gy) doses of radiation, an immune-modulating chemotherapeutic agent (temozolomide), and an immune checkpoint inhibitor, durvalumab. Both agents combat cancer in different ways, and their effects when combined with radiation have been a significant area of study [23,24,30,31,32,33,34,35,36]. We used a state-of-the-art automated and cloud-based clonogenic assay (CytoSMART Omni, Eindhoven, The Netherlands) to measure cell survival after 21 days of treatment. We quantified cell migration using a commercially available Electric Cell Impedance Sensing device (ECIS, Applied Biophysics, New York, NY, USA). Our in vitro assays deliberately left out the full immune-system-level responses in order to focus on responses to anticancer agents within and between cancer cells themselves. Such assays, with relatively few external forces, have been the bedrock of radiobiological advances for decades.

Our results showed that T98G and U87 MG treated with radiation migrated significantly more than untreated cells (*p* < 0.05), while treated T98G cells also expressed depleted cell–cell adhesion (*p* < 0.05), both of which indicate the possible inadvertent enhancement of local invasion potential in cells prior to cell killing by radiation therapy. Treatment with temozolomide further magnified these effects (*p* < 0.01) in T98G, showing the possible pro-metastatic effects of temozolomide when used in combination with radiation therapy for glioblastoma. These putative pro-metastatic or pro-invasive effects may negatively impact long-term treatment outcomes, and since the combination of radiation and temozolomide feature in the standard of care for glioblastoma, there is an urgent need for more effective antimetastatic strategies against glioblastoma. However, treatment with durvalumab did not significantly affect cell migration, and, at the sublethal radiation dose, T98G cells showed restored cell–cell adhesion (*p* < 0.05) compared to solely irradiated conditions, indicating a potential antimetastatic effect of durvalumab. Surprisingly, both agents showed little effect on cell survival at both sublethal and lethal doses for both glioblastoma cell lines. In testing, T98G cell survival was most affected by radiation (*p* < 0.0001), which dominated the cell-killing effect of treatment. Therefore, TMZ and durvalumab may not be effective agents in tumor control via direct effects on cells and, instead, may amplify tumor control mainly in the presence of the immune system, which stresses the immunotherapeutic potential of therapy using these agents.

Using in vitro assays in multimodal, multiparametric tests, we have characterized interactions between the immune-modulating agent, TMZ, and the immune checkpoint inhibitor, durvalumab, used in radioimmunotherapy for glioblastoma. We detected changes to cell behavior and characteristics which may have otherwise been difficult to detect in vivo but may have impacts on long-term treatment outcomes. Therefore, our assays may corroborate or provide mechanistic insights about results of current in vivo testing, including phase I and II clinical trials which involve these agents in therapy for glioblastoma [24,37]. In the long term, methods developed in this work may serve as a basis for the development of patient-specific targeted therapy, which may allow for the testing of multimodal therapies against one another for patient cells to help determine optimal treatment strategies.

## 2. Materials and Methods

### 2.1. Cell Lines, Culture Methods, and Cell Preparation for Experiments

Cell lines used for this research were the glioblastoma multiforme cell lines, T98G (ATCC CRL-1690) and U-87 MG (ATCC HTB-14), both purchased from the American Type Cell Culture Collection (ATCC, Manassas, VA, USA). T98G cells have a fibroblast morphology, while U87 have an epithelial morphology. Both cell lines were cultured following the same general protocol from ATCC. Briefly, both GBM cell lines were cultured in Eagle’s Minimum Essential Medium (EMEM, ATCC 30-2003) or Dulbecco’s Modified Eagle Medium (DMEM, Corning 10-013-CMR) culture medium supplemented with 10% fetal bovine serum (FBS, Gibco, New York, NY, USA, 10100147) and 1% penicillin–streptomycin (P/S, Sigma Aldrich, St Louis, MI, USA, P4333-100ML). Cells were grown in consistent medium for each set of trials to reduce the influence of the culture medium on experiments. When switching formulations, cells were given ample time to acclimate to the new medium before use in experiments. Cells were seeded in either T-25 or T-75 filtered cell culture flasks (Fisher) at a density of $1.0\times 10^{5}-2.0\times 10^{5}$ cells/mL (Figure S1). Cells were grown to a density up to $6.0-8.0\times 10^{5}\;$ cells/mL before splitting and re-seeding at a 1:3 to 1:6 ratio. Experiments were conducted at a standard rate of $1.0\times 10^{6}\;$ cells/mL cell density, except for clonogenic assays, which required a much lower seeding density on the order of 50–5000 cells/mL.

A flask to be cultured first had its old medium discarded, and then the flask was washed with an appropriate volume of phosphate-buffered saline (PBS, Gibco 20012027) to remove all old medium, which was then discarded. Next, cells were lifted with 0.25% trypsin–EDTA (Gibco) and incubated for 5–10 min. Once cells had detached entirely from the bottom of the flask, the trypsin was neutralized with twice the volume of culture medium as the volume of trypsin added. Then, 90 µL of the resulting solution was removed and placed into a separate tube containing 10 µL trypan blue (Sigma-Aldrich, St Louis, MI, USA) for counting of cell viability by hand on a hemocytometer or Invitrogen Countess II automatic cell counter (Thermo Fisher Scientific, Waltham, MA, USA, AMQAX1000). The remaining cells and neutralized trypsin solution were then placed in a centrifuge run at 800 RPM for 5 min. Once finished, the solution was carefully removed without disturbing the pellet of cells at the bottom of the tube. The cells were then resuspended in fresh complete culture medium by gently pipetting to ensure homogeneity and seeded in flasks at appropriate dilutions. All chemicals used in cell culture were pre-warmed in a water bath to 37 °C to avoid temperature shock. Preparing cells for use in experiments followed the same protocol as in cell culture (Figure S1), but the final concentration of cells differed by experiment, as previously noted. Cell cultures for experiments were only used if viability by trypan blue cell count met or exceeded 92%. Cells were ensured to be in the logarithmic phase of cell growth prior to use in experiments. Cells treated with outside agents, including chemotherapeutic drugs, were incubated for 30–45 min following initial exposure before data collection or irradiation, unless otherwise noted, to ensure metabolism and/or uptake into cells as desired.

### 2.2. Drug Dosage and In Vitro Treatment of Cells

Temozolomide (Sigma T2577-25MG) was measured out following the guide by Liston and Davis (2017), which lists the in vivo dosage corresponding to a standard clinical dose of anticancer drug [38]. For temozolomide, a typical standard dose for GBM patients is 150 mg/m^2^ patient, which, in vitro, corresponds to a 7300 ng TMZ/mL solution [38]. The guide aims to reduce the commonality of overdosage in vitro, which is difficult to control and nontrivial to calculate due to various biological and physical differences between in vitro and in vivo systems [38]. Such overdosage issues, particularly with temozolomide in the treatment of glioblastoma, include the absence of the blood–brain barrier in vitro, and the absence of biological clearance of the drug. The net effect of these differences and others results in frequent overdose in vitro, which has limited utility in determining clinically relevant results [38]. For many anticancer drugs, there are effects which appear or disappear at high dose, many of which are clinically unachievable [38]. Therefore, reporting these effects has limited utility. With our aim to evaluate and investigate clinically relevant treatments, we followed the guide to ensure maximum translation between our in vitro testing and the clinic. We used a stock concentration of 0.8 ± 0.42 mg/mL solution. According to the guide, a 150 mg/m^2^ standard in vivo dose of temozolomide corresponds to a concentration *C_max_* of 7300 ng TMZ/mL solution in vitro [38]. Using our stock solution, our final concentration for the corresponding clinical dose was 7304 ± 523 ng TMZ/mL. By linear extrapolation, the limits of error fell well within the maximum and minimum dosages used in the clinic for both concurrent and adjuvant TMZ with radiotherapy, at 3650 ng TMZ/mL minimum and 9733 ng TMZ/mL maximum doses, corresponding to 75 and 200 mg TMZ/m^2^ patient in vivo doses [39,40]. Therefore, our doses of TMZ were kept within clinically relevant margins.

Effective, clinically relevant in vitro concentrations of durvalumab (Fisher MedChemExpress HY-P9919) are generally not as well standardized as those for temozolomide, as some clinical trials are still in progress for determining the proper dosage for patients in various cases [37]. Testing has revealed the 50% effective dose (EC50) at 7.64 ng durvalumab/mL (0.0522 nM) [41] and the manufacturer quotes a half-maximal inhibitory concentration (IC50) of 0.1 nM, approximately double the EC50 dose. Based on the recommendations of Liston and Davis [38,42], in testing, concentrations were kept below 10 times the IC50 to avoid effects outside of clinically relevant doses. Preparation of the doses of durvalumab mirrored that of the temozolomide; however, per recommendations from the manufacturer, stock solutions were not stored for extended periods and the undiluted solution was kept at 2–4 °C to avoid antibody deactivation. Additionally, stock solutions were diluted in PBS (pH 7.2, Gibco) as recommended.

### 2.3. Irradiation of Cells: Faxitron CellRad

Radiation was delivered in-lab via the Faxitron (Tucson, AZ, USA) CellRad compact X-ray system (Figure S2). The machine delivers X-rays at a tube potential from 10 to 130 kVp and a tube current of 0.1–5 mA, to a field size from 9 to 27 cm diameter (40° beam divergence) depending on the level of the turntable tray. The CellRad can achieve maximum dose rates of 45 Gy/min for an unfiltered beam and 8 Gy/min with a 0.5 mm Al filter. In our trials, our dose rates typically measured between 0.500 Gy/min and 0.650 Gy/min. The machine has a built-in ionization chamber housed under the center of the turntable holding specimens, which measures the dose rate and accumulated dose to the sample in the field, allowing us to use the “Auto-Dose Control” feature when irradiating the samples. Auto-Dose Control (ADC) allows users to input a selected dose (Gy) and toggle kVp and tube current settings. The machine irradiates samples until it reaches the required accumulated dose. The machine also has two other modes: Manual Mode and Dose Mapping Mode. Manual Mode (Timed Control) allows users to select a specified period for samples to be irradiated over, with their selected kVp and mA settings. Likewise, with Dose Mapping Mode, the user selects kVp and mA settings and measures the dose in the chamber with the built-in dosimeter. In this mode, kVp and mA settings can be adjusted during irradiation, whereas in ADC and Manual Modes, these settings are locked in the duration of irradiation. For our experiments, we used the Auto-Dose Control mode throughout (Figure S2), with settings at 100 kVp, 5 mA tube current, and 26.8 cm field size to irradiate our cells. At this field size, our source to surface (platform) distance was 38.4 cm. To ensure uniformity of dose across experimental conditions, all irradiated specimens were placed in microtubes or flasks central to the beam axis. As the beam covered a large field size relative to our sample size, all conditions were placed within an acceptable region of uniformity in the beam. All samples were placed on shelf 1 or the furthest shelf (Figure S2), to use the largest field size and achieve the best uniformity of beam profile for our irradiated regions. Prior to each experiment involving the CellRad, we allowed the machine to warm up for 30 min and allowed the machine to perform its built-in routine dose quality assurance. This automated quality assurance program ensures that the X-ray machine and on-board dosimeter are performing as intended.

### 2.4. Electric Cell Impedance Sensing (ECIS) for Migration Measurement

The Electric Cell-Substrate Impedance Sensing (ECIS) device is commercially available from Applied Biophysics (New York, NY, USA) (Figure S3). The ECIS is a noninvasive, robust device for electrically measuring a variety of cell characteristics, including morphology, proliferation, and migration [43,44]. The ECIS is a well-established, noninvasive mode of detecting changes in cell characteristics over extended periods, having been used in several studies, by others and by ourselves [45,46,47]. The primary focus of our work with the ECIS was an evaluation of possible pro- or antimetastatic effects of treatment. Because many anticancer agents, especially chemotherapeutic agents, were designed to combat cell proliferation rather than metastasis specifically, other unintended effects involving metastasis may affect the efficacy of treatment. Because metastasis is the primary factor contributing to cancer lethality, an evaluation of cell behavior and migration following treatment is of great importance.

The complete ECIS setup includes an incubator (here, Fisher MIDI-40), array station housed in the incubator, station controller, and computer. The incubator used for the ECIS was kept at 37.0 °C and 5.0% CO_2_. The array station inside the incubator held up to two cell arrays (Figure S4) to be measured and housed various electronics and signal processing circuits for data acquisition. The station was connected by a flat wire to the station controller (Zθ) outside the incubator, which was connected to a computer via USB. The station controller housed most of the critical electronics, including the variable frequency alternating current source used to perform impedance measurements.

Impedance in resistor–capacitor circuits generally follows the simple relation

(1) $$X_{c}=\frac{1}{2\pi fC^{\prime }},$$

(2) $$Z=\sqrt{R^{2}+X_{c}{}^{2}}.$$

$X_{c}$ is the capacitive reactance, *Z* represents the overall impedance, *C*’ is the equivalent capacitance, and *R* is the resistance of the circuit. Since the capacitive reactance is dependent on the frequency of the AC current, its influence on total impedance can be selectively modulated. The ECIS system measures the complex impedance of the overall circuit and one may reconstruct the resistance and capacitance from the impedance data.

The variable frequency AC source allows for the selective focus of data, where a lower frequency results in a higher capacitive reactance (Equation (1)), dominating the overall impedance measurement (Equation (2)). Conversely, a higher AC frequency lowers the capacitive reactance, allowing for the selection of resistance-dominated impedance readings, which better isolates resistance-coupled measurements.

These measurements are processed and displayed in the Applied BioPhysics program on the computer. The system is capable of measuring the resistance, capacitance, and impedance of each well of the array with minimal electrical noise, which yields information about cell migration, viability, and other biological and biophysical properties [45,46].

We used 8W10E+ arrays from Applied BioPhysics (Figure S4). They feature 40 gold-plated, 1.96 mm^2^ electrodes in each of the eight wells. They are capable of measuring 2000–4000 cells in a confluent layer at a well volume of 600 µL. The main advantage of this assay over others is a higher relative number of measured cells, which smooths statistical fluctuations, yielding cleaner data. The experimental setup of the ECIS followed the process outlined in Figure S5. Prior to inoculation with experimental conditions, the array wells were filled with cell-free medium as a test medium and inserted into the array holder for connection tests and stabilization. Following checks and stabilization, the array was removed and the medium discarded. After preparing the experimental conditions, the samples were loaded into the wells in order (Figure S5, steps 3, 4), allowed to stabilize for 15 min, and “checked” again to ensure electrical connection with the experimental conditions in place. Following final checks, the experiment was initiated.

As cells spread to cover the bottom of the well, the resistive flow of current under and between cells changes. Additionally, the capacitively coupled flow through individual cells in the layer contributes to the overall impedance of the system. However, extracting parameters which describe specific characteristics of cell behavior, such as migration, is not straightforward. Because the ECIS measures the sum of the influences of all cell behaviors and characteristics on the impedance, data extraction required extensive modeling.

Giaever and Keese, the inventors of the ECIS system, also pioneered ECIS modeling to extract meaningful parameters from raw ECIS data [48,49]. Their model of a confluent cell layer approximated the cells as cylinders with insulating membranes, filled with a conductive electrolyte [48,50]. In this model, the main electrical influences of the cell layer on the overall impedance could be split into three respective current flows. The first is the basal current, capacitively coupled with the second, the apical current, which travels “through” the cell layer vertically with respect to the electrode plane [48]. The third is the paracellular current, also called the resistive flow, which describes the current flowing underneath the cell layer along the plane of the electrode array before permeating the cell layer through tight junctions between cells [48]. The ECIS system enables the isolation of each current, which allows users to selectively view resistively coupled or capacitively coupled effects [48].

Giaever and Keese’s “disc model” of ECIS operates under four core assumptions [50]: (i) the potential above the cell layer, *V_m_*, is constant over the course of the experiment; (ii) the paracellular current flow is assumed to originate from a point underneath the center of each cell, traveling a distance equal to the radius of the cylinder before reaching the tight junction; (iii) the cell membrane does not contribute to the resistive flow of current and is therefore purely capacitive in the effective circuit; (iv) the resistance and capacitance of the electrodes in the array remain unchanged. The main result of the disc model is the equation for the total impedance *Z_c_* across the range of frequencies used in measurement [50]:

(3) $$\frac{1}{Z_{c}}=\frac{1}{Z_{n}}\left[\frac{Z_{n}}{Z_{n}+Z_{m}}+\frac{\frac{Z_{m}}{Z_{n}+Z_{m}}}{\frac{i\left(\gamma r_{c}\right)}{2}\frac{I_{0}\left(\gamma r_{c}\right)}{I_{1}\left(\gamma r_{c}\right)}+2R_{b}\left(\frac{1}{Z_{n}}+\frac{1}{Z_{m}}\right)}\right],\;$$

where *Z_c_* is the total impedance across the many frequencies taken, *Z_n_* is the impedance of the bare electrode, *Z_m_* is the specific impedance through the cell layer, *ρ* is the resistivity of the tissue culture medium, *r_c_* is the disk cell radius approximation, *h* is the vertical distance from the electrode to the cell, *I*_0_ and *I*_1_ are Bessel Functions of the first kind, of order 0 and 1, respectively, and *R_b_* is the barrier function, which measures the junctional resistance between cells in a monolayer per unit area. $\gamma r_{c}$ takes the form

(4) $$\gamma r_{c}=r_{c}\sqrt{\frac{\rho }{h}\left(\frac{1}{Z_{n}}+\frac{1}{Z_{m}}\right)}=\alpha \sqrt{\left(\frac{1}{Z_{n}}+\frac{1}{Z_{m}}\right)}.$$

In Equation (4), alpha (α) and the barrier function *R_b_* are the main parameters of interest for cell adhesion and attachment studies, and tight junction (TJ) studies, respectively [50]. Briefly, the parameter alpha corresponds to the constraint of current flow beneath the cell layer. A measurement of alpha thus yields information about the size of cells and height of the cell layer above the substrate for attachment studies [48]. The barrier function, *R_b_*, has long been the main focus of ECIS studies focusing on tight junction dynamics [48]. TJ function is critical to cancer metastasis in the tumor, where compromised TJ function may allow cells to break away from a primary tumor into the surrounding tissue, particularly epithelial tissue, which cancer cells infiltrate in the metastatic cascade [48]. The TJ is the first critical structure impeding cell invasion and is thus a critical measure of the effects of treatments on cancer metastasis.

**Figure 1:**
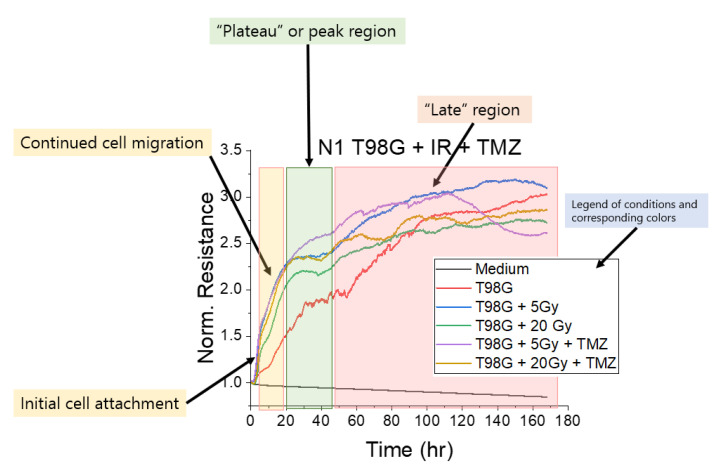
Typical ECIS plot of normalized resistance (64,000 Hz). Cells begin by adhering to the bottom surface of the array, forming an initial buildup region. After initial attachment to the substrate, cells migrate toward the preferred distribution. For T98G, cells migrated to cover the available space in the array, increasing resistance until reaching a peak, dubbed the “plateau region,” at which time cells have reached maximal coverage. The late region is characterized by continued proliferation and/or cell death, which increases or decreases resistance, respectively.

The general shape of ECIS resistance plots (Figure 1) includes an initial buildup region where cells suspended in solution begin to adhere to the bottom surface of the arrays, followed by a region of cell migration, which increases (T98G) or decreases (U87) resistance and impedance until reaching a peak (or trough), called the “plateau region”. At this timepoint, cells generally have reached their preferred distribution, and further changes to resistance are due to cell death or further proliferation, which was seen in T98G trials. However, it was found that some cell lines, including U87 MG used in this work, tend to preferentially adhere to one another rather than some substrates at high cell density, forming neurospheres [51,52], manifesting in significantly different plot characteristics than for monolayer-forming cells. Interpretation of the U87 ECIS data was thus less straightforward than in the T98G trials due to the cells’ tumorigenicity. Using the shape of the ECIS plots, we determined several measures and parameters, corresponding to relevant cell characteristics for analysis. It should be noted that the general shape or features of the ECIS plots were reproducible in a cell-type-dependent manner and that the time to reach these features occurred within ranges, rather than at exact timepoints.

The late migration parameter, *LR*, the measured resistance following the peak region, gives insight into the late effects of treatment on cell survival. Following the peak region, where cells have completed initial migration toward their preferred distribution, increasing resistance in monolayer-forming cells correlates with continued proliferation and increased coverage, whereas decreasing resistance is linked to cell death, as dying cells lift from the array surface. The second key parameter, *ROM*, represents the apparent rate of migration. *ROM* was calculated by measuring the slope of the normalized resistance in the approximately linear region of initial migration. To smooth fluctuations and micromotion effects, the average slope was calculated for the largest time range possible within the approximately linear region of migration.

**Figure A1:**
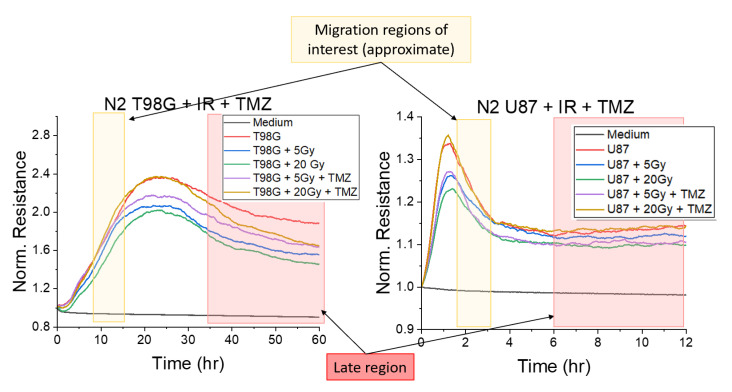
Choice of regions of interest for rate of migration (ROM) and late resistance (LR) analysis of T98G and U87 following treatment. Time frames used for both T98G and U87 were selected for each experiment and rested consistently within the migration region for all trials. The cell behavior was considered in the selection of the migration region. Timescales shown were shortened for ease of visualization.

**Figure A2:**
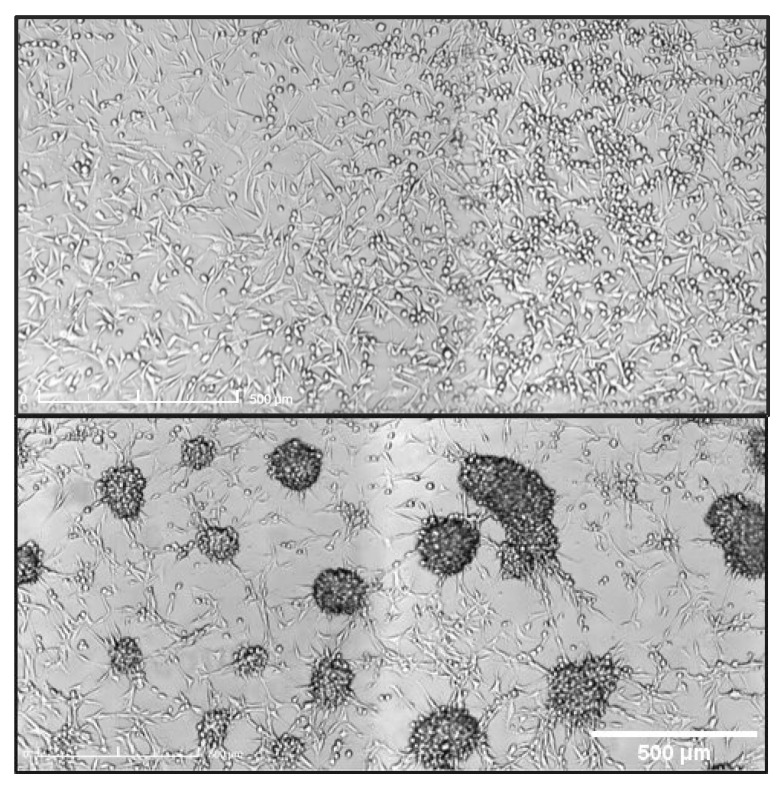
U87 MG seeded at high density ($5.0\times 10^{5}$ cells/mL), in a 24-well plate, demonstrating preferential cell–cell adherence. Images captured on the CytoSmart Omni system. **Top:** Cells 2 h after initial seeding. Cells have begun adhering to the bottom surface of the culture assay and have already started migrating. **Bottom:** Cells 24 h following initial seeding. Cells have migrated significantly, preferentially adhering to one another to form neurospheres. Notice also that there is significant empty space on the bottom surface of the well, showing that cluster formation is preferred in some aspect over formation of a cell monolayer for U87 MG cells, leading to reduced resistance with migration, in ECIS, following initially increased resistance (Figure A1). It should be noted that imaging of the ECIS array at 168 h post-treatment showed slightly smaller cluster size and increased array coverage in treated cells as compared to untreated cells.

Selection of higher frequencies for analysis reduced the influence of capacitance on measurements, isolating the effect of resistance on the impedance for the analysis of *ROM* and *LR*. This was favorable because capacitance is generally less indicative of cell migration, while resistance is more directly dependent on array coverage and, therefore, movement. Frequencies above 40 kHz have been suggested as best suited to following changes in electrode coverage due to cell spreading [46]. For *ROM* calculations, we isolated the region of the plot corresponding to initial cell migration toward the preferred distribution following attachment. The region of interest was in the linear region approaching the plateau (Figure A1). For T98G and U87 analysis, the region of interest rested early in the experiment. The key difference in analysis for the two cell lines was that the monolayer-forming T98G cells migrated to cover the array surface, which manifested in increasing resistance, while the tumorigenic U87 tended to migrate to form clusters, which decreased resistance (Figure A1 and Figure A2). The calculation for the defined parameter, *ROM*, was a simple slope calculation (Equation (5)):

(5) ROM=Rnorm cond at tb−Rnorm cond at tatb−ta · ΔRnormunitlesshr,

where $R_{norm\;cond\;at\;t}$ is the normalized resistance measurement at time *t* for a given condition, *t_b_* is the selected endpoint of the period of interest, and *t_a_* is the selected starting point of the period. We defined the rate of migration as measured by resistance as the change in the normalized resistance (dimensionless) over the change in time. *ROM* is thus more specifically the rate at which the resistance changed in the period associated with initial migration. The normalized resistance (Equation (6)) was calculated by dividing the measured resistance at time *t* by the resistance for the same condition measured at time “zero”, the first measurement in the series. This accounted for differences in connectivity between arrays and gave a better translation of data between trials for direct comparison.

(6) $$R_{norm\;cond\;at\;time\;t}=\frac{R_{cond\;at\;t}}{R_{cond\;time\;“0”}}.$$

Following the plateau region, cells continued to proliferate or underwent cell death, which increased or decreased resistance (and, therefore, impedance), respectively. The late resistance, *LR*, quantified this late cell death by averaging the resistance in the region following the peak. Using these data, we quantified the impacts on cell survival for each treatment in the experimental conditions by calculating the percent difference %Δ in the mean normalized resistance for each condition relative to that of the untreated condition over all timepoints following the initial plateau (denoted time *p*) through to the final timepoint at 168 h, $\bar{LR}$ (Figure 1 and Figure A1), calculated using Equation (7):

(7) $$\bar{LR}=\frac{\sum_{t=n_{p}}^{n_{168}}(R_{cond})}{\left(n_{168}-n_{p}\right)\times R_{t=0}},$$

where the sum of the measured resistance following the initial plateau (sum of resistances at timepoints $t=n_{p}\;to\;t=n_{168}$; *n* is the number of the corresponding timepoint) is divided by the number of timepoints to calculate the mean resistance over the period, and the resistance measured at the first timepoint, *t* = 0 for normalization, yielding the normalized resistance for each condition over the period following the initial plateau.

For a cell monolayer, cell death resulted in decreased resistance as cells lifted from the bottom surface of the cell array. This was the primary motivation for the use of *LR* as a parameter representing late cell death. However, as previously indicated, raw ECIS data represent the combined effects of cell migration, proliferation, attachment, and other behaviors or physical characteristics, which makes *LR* a general representation of the net trends following the peak region. It therefore does not represent solely cell death. However, as a general parameter, it provides valuable insight into late effects of treatment over time. To measure cell death most directly, we instead focused on clonogenic assays, which are the gold standard in cell survival assays. Data from clonogenic assays were valuable in aiding the interpretation of ECIS data, and because cell death is a continuous process, this allowed for more concrete isolation of the effects of treatment on cell survival over an extended period. In combination with the ECIS data, which provided valuable insight into cell migration, morphology, and metastasis, clonogenic assays built up a multidimensional and multiparametric analysis of the cellular-level effects of treatments for glioblastoma.

### 2.5. Clonogenic Assays

Clonogenic assays are the gold standard in measuring cell survival following treatments. Clonogenic assays selectively detect surviving cells which retained the ability to undergo unlimited reproduction [53]. This proliferation of surviving cells results in the formation of colonies of 50 cells or more, which are then counted. Provided that the user knew the number of cells plated initially, the survival fraction could be calculated by counting colonies, since each cluster was assumed to have originated from a single cell for a small enough number of plated cells. Because radiation damage frequently results in a loss or inhibition of cell reproduction, clonogenic assays give a good measure of cell survival and therefore efficacy of treatment. They are regarded as a straightforward, clear evaluation of cell survival following treatment, and information taken from these assays is widely used to inform treatment plans in oncology [54].

**Figure A3:**
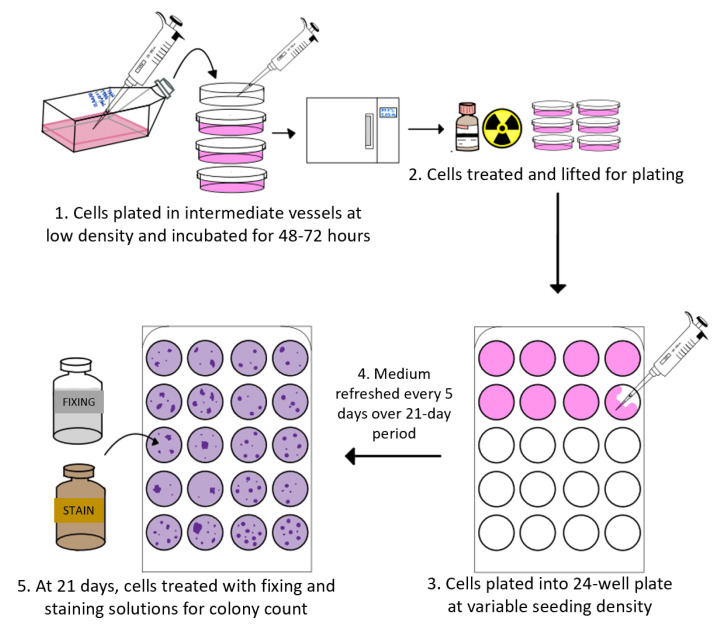
Preparation of clonogenic assays. Step 1: Cells were taken from culture vessels and seeded in intermediate vessels at reduced cell density to ensure count consistency with the variable seeding strategy. Following seeding, cells were incubated for 2–3 days prior to treatment to allow for recovery. Step 2: Cells were treated and lifted for plating. Step 3: Cells were plated into clonogenic assay well plates at the variable seeding density. The plate was placed into the incubator for the duration of the experiment. Step 4: Medium was refreshed every 5 days for the duration of the experiment to ensure ample access to nutrients. Step 5: At the endpoint, cells were treated with fixing and staining solutions for final manual colony counts.

We carried out two clonogenic assays, manual and automated, on each experimental cohort following provided manufacturer protocols as well as the protocol formulated by Franken et al. [53]. Some minor adjustments were made to the original protocols to adapt to the materials used. Cells were grown and harvested in the logarithmic phase of growth for seeding. In the process, approximately 50–5000 cells were seeded in each well of a 24-well plate depending on the expected cell survival, in a final volume of 0.6 mL of solution, with two wells of each experimental condition prepared in a 24-well plate (Figure A3). All cells were treated prior to plating. Cells were cultured as normal, but prior to seeding in the well plate, to ensure accuracy of cell counts, we plated cells in intermediate, smaller culture wells. We used 35 mm diameter plates (Falcon 353001) treated for cell culture for each condition as the intermediate vessel, in which cells were allowed to grow for 2–3 days prior to treatment. Using these vessels, we could treat individual conditions and perform lifting and seeding separately. Alternatively, lifting cells prior to treatment risked compromising cell counts due to the attachment of adherent cells should preparation and treatment take a sufficiently long time. Thus, we ensured seeding of viable cells with better accuracy of cell counts. Following treatment, lifting, and centrifuging, cells were resuspended and seeded at proper densities in well plates, diluted in 0.6 mL of medium per well. Following seeding, plates were placed on the CytoSmart Omni system for imaging. Medium was refreshed every 5 days throughout the experimental period, and cells were allowed to grow for 21 days. When seeding the assays, a variable seeding strategy was necessary to ensure acquisition of meaningful data. This enabled us to balance seeding few enough cells to avoid overlaps in colony formation so that colonies each originated from single cells, while still allowing us to detect surviving cells in especially lethal or high-dose conditions. After several iterations, the optimal variable seeding strategy that we found was as follows: 0 Gy, 50 Cells; 2 Gy, 100 Cells; 5 Gy, 500 Cells; 10 Gy, 1000 Cells; 20 Gy, 2000 Cells; and 50 Gy, 5000 Cells.

**Figure A4:**
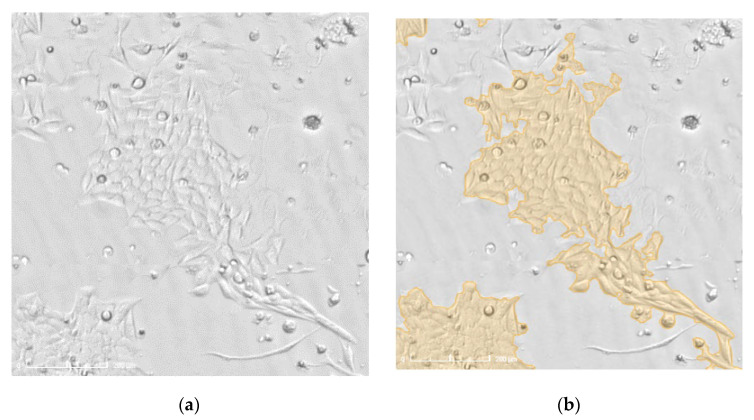
**Images of T98G cells taken on the CytoSmart Omni.** (**a**) T98G cells in a typical Omni image in brightfield viewing mode. (**b**) T98G cells under colony viewing mode. Colonies of sufficient size are displayed in the orange highlight and were included in colony analysis.

At the endpoint, for the manual counting technique, cells in assays were fixed and stained using a BioPioneer CellMAX^TM^ Clonogenic Assay Kit. Colonies were then counted under a microscope. Additional independent automated analysis was also done by the cloud-based CytoSmart Omni system. The CytoSmart Omni is a cloud-based image analysis tool used for multiple types of cell analysis (Figure A4). The system scans high-resolution images of the plate or assay loaded onto the surface of the scanner. It can be programmed to scan the plate over set time intervals up to 24 h between automated scans, which allows for the use of multiple endpoints. We used the built-in colony detection analysis (Figure A4, right), which counted the number of colonies formed automatically. Well plates were loaded onto the Omni following treatment for imaging at 24-h intervals.

From the assay data, we calculated cell survival fractions using the clonogenic assay equations (Equations (8) and (9)). Colonies were counted manually in addition to the colony analysis done on the Omni, where a colony is a group of at least 50 cells.

(8) $$Surviving\;Fraction\;\left(SF\right)=\frac{no.\;of\;colonies\;formed\;following\;treatment}{no.\;of\;cells\;seeded\;\times PE}.$$

(9) $$Plating\;Efficiency\;\left(PE\right)=\frac{no.\;of\;untreated\;colonies\;formed\;}{no.\;of\;untreated\;cells\;seeded\;}.$$

The surviving fraction was plotted as a function of the dose to produce the survival curve, which showed the surviving fraction of cells following treatment. The survival fraction is corrected by the plating efficiency, *PE*. The plating efficiency is a normalizing factor which accounts for cell death due to stresses in preparation and plating by counting the number of untreated cells which survived over the course of the experiment. By correcting *SF* with the plating efficiency, we better isolated the effect of treatment on cell survival rather than death due to environmental stresses. Both the plating efficiency and the surviving fraction assume that each colony formed originated from a single cell. Careful consideration of the number of cells plated was necessary to strike a balance between statistically sound survival data while satisfying the key assumption.

The corrected survival fraction was then fit to a cell survival model. We assumed that our cell lines and survival data were described by the linear-quadratic (LQ) model of cell survival:

(10) $$SF=e^{-\alpha D-\beta D^{2}},$$

where *D* is the dose of radiation, and *α* and *β* are parameters describing the cell’s sensitivity to radiation-induced cell death due to DNA double-strand breaks from a single ionization event and two separate events, respectively. By determining the *α*/*β* ratios and the individual parameters for our treatments, we could then describe the sensitivity of the cells to treatment and changes to sensitivity with the addition of other treatments, which helped to probe therapeutic windows relevant to therapy.

### 2.6. Statistical and Error Analyses

Robust statistical analysis was performed using analysis of variance (ANOVA) in Origin (OriginLab, Northampton, MA, USA) to determine the statistical significance of findings for measured parameters. ANOVA is a parametric method for means-based comparison of data groups. A major benefit of Origin’s ANOVA algorithm is that it minimizes the probability of type-I error in statistical analysis, in which the null hypothesis is wrongly rejected, causing users to wrongly conclude that results were statistically significant. Error analysis was performed using the standard error of the mean (SEM). We performed at least three independent repeats of every experiment (*N* = 1, *N* = 2, *N* = 3). We report both the representative independent repeats and the averages of the triplicate experiments.

For both *ROM* and *LR*, it was useful to calculate the percent difference between conditions for each trial (N1, N2, N3), to better visualize changes due to treatment and to normalize to normal cell behaviors. The percent difference averaged over three trials, $\bar{\%\Delta }$, was calculated as shown in Equation (11), where *P* is the parameter of interest, with the standard error of the mean as the uncertainty:

(11) $$\bar{\%\Delta }=\frac{1}{3}\left[\frac{P_{treated\;N1}-P_{untreated\;N1}}{P_{untreated\;N1}}+\frac{P_{treated\;N2}-P_{untreated\;N2}}{P_{untreated\;N2}}+\frac{P_{treated\;N3}-P_{untreated\;N3}}{P_{untreated\;N3}}\right]\times \;100.$$

## 3. Results

### 3.1. Results Follow from Hypotheses

With the goal of developing in vitro assays for advancing radioimmunotherapy against brain tumors, we posited the following main research question: what are the cellular effects of immunotherapeutic agents alone and in combination with chemotherapy or radiotherapy against glioblastoma? This led to the following three sub-hypotheses: treatment of glioblastoma cells with the immune-modulating chemotherapeutic agent, temozolomide, and the immune checkpoint inhibitor, durvalumab, with radiation, (i) alters cell migration in distinctive ways that implicate metastasis; (ii) compromises cell–cell adhesion, providing distinguishable readouts of local invasion potential, and (iii) causes increased cell death with clonogenic signatures. The following results follow from these hypotheses.

#### 3.1.1. Treatment with Radiation and Concurrent Temozolomide Increases Cell Migration Relative to Radiation Alone

**Figure 2:**
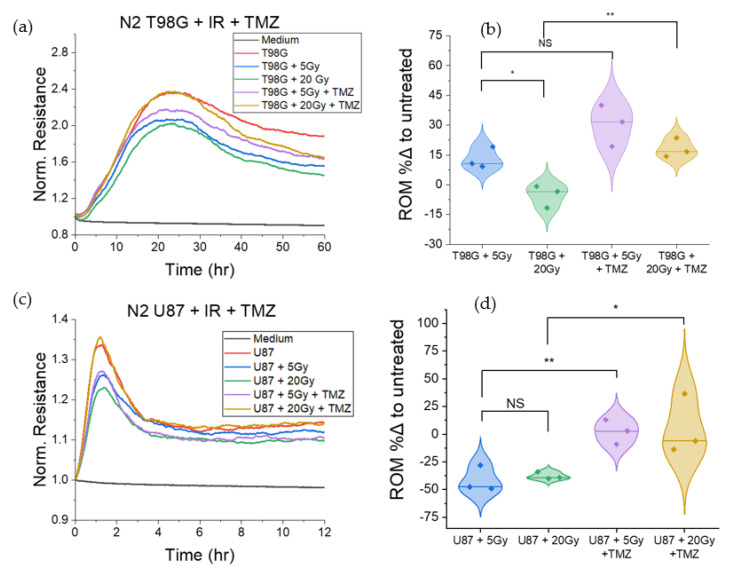
ECIS migration of T98G and U87 glioblastoma cells treated with radiation and temozolomide at 7300 ng/mL. Representative plots of normalized resistance in the first 60 h (**a**) and 12 h (**c**) post-treatment for T98G and U87, respectively, are shown. Calculated percent differences in *ROM* for treated conditions, relative to the untreated control, for T98G (**b**) and U87 (**d**). * *p* < 0.05, ** *p* < 0.01, NS non-significant.

ECIS is particularly useful for visualizing cell migration in real time following treatment. Using the previously defined metric of cell migration, *ROM*, taken relative to the untreated condition, ECIS results revealed that T98G cells treated with 20 Gy of radiation and 7300 ng/mL TMZ migrated more (*p* < 0.01) than cells treated with 20 Gy alone. In contrast, at the 5 Gy dose level, T98G cells treated with TMZ did not migrate significantly more than cells treated with radiation only, indicating changes to cell migration at the 20 Gy dose level when treated with TMZ (Figure 2a,b). With radiation alone, however, *ROM* was elevated for the 5 Gy dose (*p* < 0.05) at +13.0% ± 3.1%. With the addition of TMZ, cells migrated more than the untreated condition (*p* < 0.05), but this was not significantly increased compared to the radiation-only condition at the 5 Gy dose level, with *ROM* + 30.3% ± 6.0% relative to the untreated condition. At the 20 Gy dose level, cells treated with temozolomide migrated significantly more (*p* < 0.01), with *ROM* 18.17% ± 2.8%.

U87 cells subjected to the same treatments showed increased *ROM* (*p* < 0.05) when treated with 20 Gy and TMZ as compared to 20 Gy only, and similarly increased *ROM* (*p* < 0.01) at the 5 Gy dose level when treated with TMZ versus radiation alone (Figure 2c,d). Results indicated changes to cell migration for U87 at both radiation doses when treated with TMZ. U87 cells treated with radiation only had significantly lower *ROM* as compared to the untreated condition (*p* < 0.01), at −41.6% ± 6.8% and −37.8% ± 1.8% for 5 and 20 Gy radiation doses, respectively. When treated with TMZ, *ROM* was not statistically different compared to the untreated condition, at +2.2% ± 6.4% and +5.4% ± 15.6% at 5 and 20 Gy + TMZ, respectively. With U87 cells, *ROM* results may not correlate with migration away from tumors owing to their preferential formation of microtumor-like structures, or neurospheres (Figure A2), rather than a solid monolayer, within 24 h of seeding. Thus, with decreased cell coverage of the ECIS array as cells migrate toward clusters and lift from the bottom surface of the array to form spheres of cells, the decreased *ROM* compared to the untreated condition may be indicative of increased migration toward cluster formation for U87 cells.

#### 3.1.2. Treatment with Durvalumab Does Not Significantly Alter T98G or U87 Cell Migration

**Figure 3:**
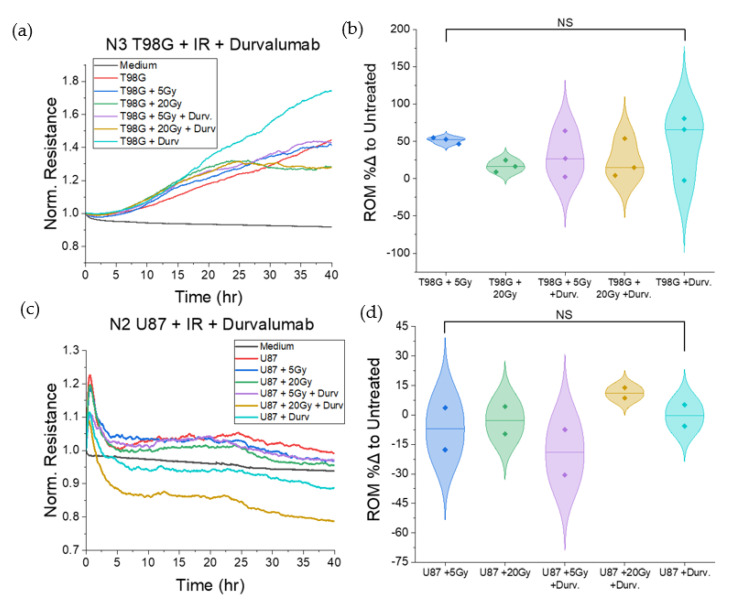
Migration-focused results of ECIS tests on T98G and U87 glioblastoma cells treated with radiation and durvalumab. (**a**) Representative plot of normalized resistance in the first 40 h post-treatment for T98G. (**b**) Violin plot of percent differences in ROM for (**a**), relative to the untreated T98G control. (**c**) Representative plot of normalized resistance in the first 40 h post-treatment for U87. (**d**) Violin plot of percent differences in ROM for (**c**), relative to the untreated U87 control. NS non-significant.

We performed similar experiments using ECIS for T98G and U87 cells treated with the immune checkpoint agent, durvalumab, to analyze the effect of treatment on cell migration at the 0, 5, and 20 Gy radiation doses. Results showed no significant changes to cell migration following treatment with durvalumab at any radiation dose, relative to radiation-only conditions, for either cell line. However, there was a wider spread (between 50% and 300%) in the durvalumab-treated T98G *ROM* (Figure 3) compared to the durvalumab-treated U87 *ROM* (30% to 100%).

#### 3.1.3. Treatment with Concurrent TMZ Marginally Changes T98G Barrier Function Relative to Radiation Only

**Figure 4:**
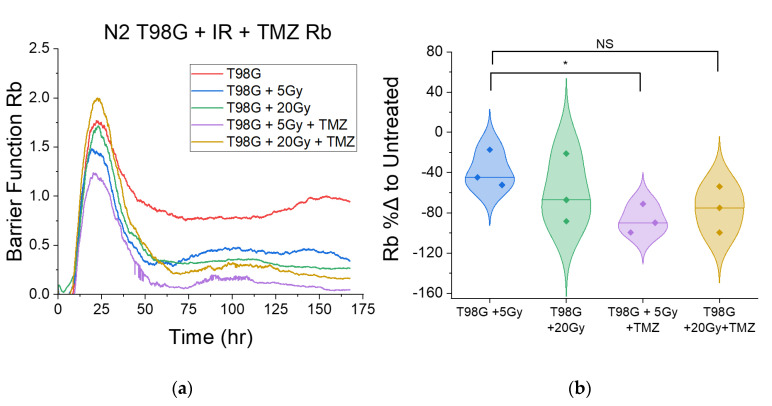
Barrier function for T98G treated with radiation and TMZ. (**a**) A representative plot of the barrier function over time. (**b**) Violin plots for (**a**) showing the direct comparison of the late barrier function relative to the untreated condition. Overall, treatment with radiation led to decreased barrier function following the plateau region. Additionally, cells treated with 5 Gy of radiation and TMZ had further decreased barrier function relative to 5 Gy only (*p* < 0.05). * *p* < 0.05, NS non-significant.

The barrier function, *R_b_*, is an ECIS model parameter representative of the retention of cell–cell adhesion in a monolayer of cells. Because loss of cell–cell adhesion is a primordial step to metastasis and promotes invasion into surrounding tissues with compromised tight junctions, analysis of the barrier function provides critical insight into potential pro or antimetastatic effects following treatment. Based on Giaever and Keese’s model of ECIS (Equations (3) and (4)), tighter cell junctions yield a higher reading of *R_b_*, whereas compromised tight junctions yield a lower *R_b_* [55]. To isolate the effects of treatment on the barrier function, measurements of *R_b_* used for comparison were of the late barrier function, which was the averaged *R_b_* in the period following the plateau region in the ECIS data. Presented are data only for treated T98G cells. Because U87 are not purely monolayer-forming cells at high density, barrier function calculations often yielded infinite or rapidly fluctuating results as clusters of cells moved over or away from electrodes and, as a result, were excluded from the modeling results. T98G cells treated with 5 Gy and 20 Gy radiation consistently showed decreased barrier function (*p* < 0.05) relative to the untreated condition (Figure 4), with percent differences ranging from −38.2% ± 10.7% to −58.9% ± 19.9%, respectively, relative to the untreated condition. With the addition of TMZ, only cells treated at the 5 Gy dose level showed further decreased *R_b_* (*p* < 0.05). Cells treated at the 20 Gy dose level showed no significant changes in *R_b_* relative to cells treated at the 5 Gy dose level for both radiation-only and TMZ-treated conditions.

#### 3.1.4. Treatment with Concurrent Durvalumab Does Not Significantly Affect T98G Barrier Function Relative to Radiation Only

**Figure 5:**
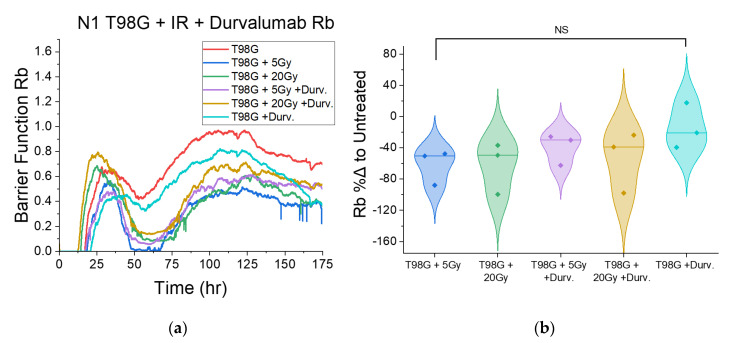
Barrier function-focused results of ECIS tests on T98G glioblastoma cells treated with radiation and durvalumab. (**a**) Representative plot of the barrier function *R_b_* post-treatment for T98G shown. (**b**) Violin plots of % differences in late *R_b_* for treated conditions, relative to the untreated control. The addition of durvalumab did not significantly affect the barrier function compared to irradiated controls. NS non-significant.

Following treatment with radiation and durvalumab, the late barrier function, *R_b_*, did not significantly change compared to irradiated T98G (Figure 5) over three trials. However, the data showed a clear spread. The first two trials, N1 and N2, showed more consistent data to each other, so, when isolating the first two trials, the results showed significantly decreased late *R_b_* (*p* < 0.05) for all 5 and 20 Gy-irradiated conditions (Figure S6). However, T98G treated only with durvalumab showed no significant change in barrier function.

#### 3.1.5. T98G and U87 Treated with Radiation and TMZ Show No Significant Change to Late Resistance Relative to Untreated Cells

**Figure 6:**
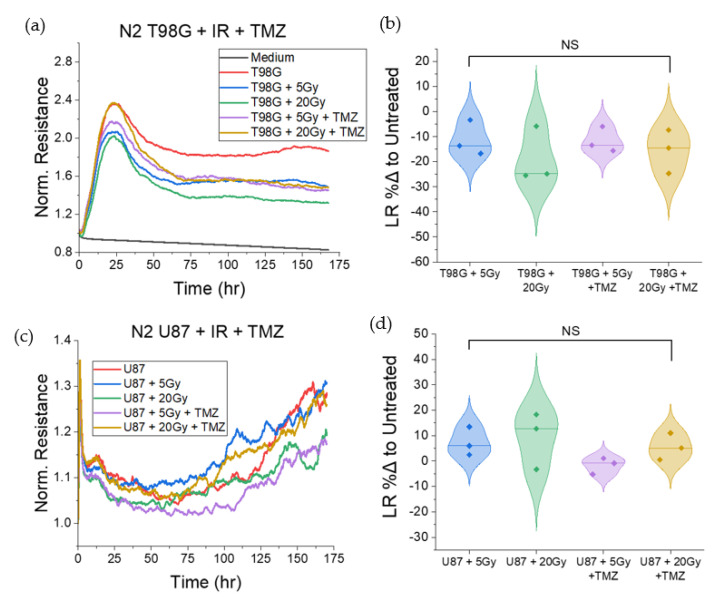
Late resistance (LR) results. (**a**) Plots of the normalized resistance over 1 week post-treatment for T98G. (**b**) Violin plots of percent difference comparisons of late resistance for treated conditions in (**a**) relative to the untreated condition. (**c**) Plots of the normalized resistance over 1 week post-treatment for U87. (**d**) Violin plots of percent difference comparisons of late resistance for treated conditions in (**a**) relative to the untreated condition. Though marginal changes can be seen in (**b**,**d**), there were no statistically significant changes to LR for either cell line treated with radiation alone or with concurrent temozolomide. NS non-significant.

The late resistance, *LR*, represents the change in resistance following the plateau region in ECIS plots. Though ECIS measurements represent several cellular-level effects under a single number, the *LR* is correlated with late effects following treatment and cell attachment. Cell survival is closely associated with *LR*, where the resistance, being related to cell coverage, decreases as dead cells detach from the substrate, for monolayer-forming adherent cells. We observed a marginal decrease in *LR* for treated T98G and a marginal increase in *LR* for treated U87 (Figure 6), though neither of these changes was statistically significant. Neither radiation nor the addition of temozolomide had statistically significant effects on late resistance in 1 week post-treatment for T98G and U87 cells.

#### 3.1.6. T98G Treated with Radiation and Durvalumab Show Decreased Late Resistance Compared to Untreated Cells

**Figure 7:**
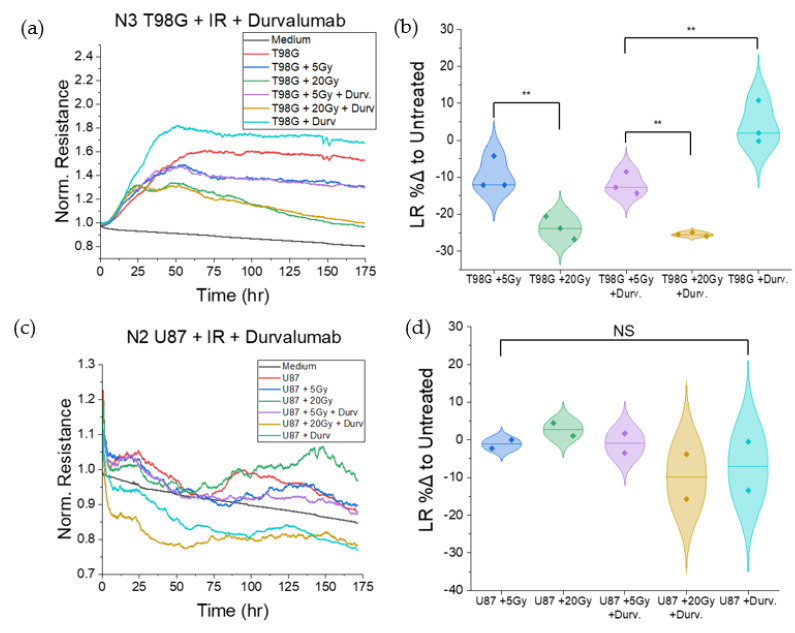
Late resistance-focused plots for T98G and U87 treated with radiation and durvalumab. (**a**) Representative normalized resistance plot for T98G treated with radiation and durvalumab over one week post-treatment. (**b**) Violin plots of comparison of late resistance (LR) between treated conditions in (**a**). (**c**) Representative normalized resistance plot for U87 treated with radiation and durvalumab over one week post-treatment. (**d**). Violin plots of comparison of late resistance (LR) between treated conditions in (**c**). The addition of durvalumab did not significantly change late resistance compared to irradiated conditions; however, cells treated with increased radiation doses showed decreased late resistance following the plateau region for T98G (*p* < 0.01). ** *p* < 0.01, NS non-significant.

T98G treated with radiation and concurrent durvalumab showed no significant changes to late resistance following treatment as compared to conditions treated with radiation only, as shown in Figure 7. However, contrary to findings in TMZ testing, for both radiation-only and durvalumab-treated conditions, escalation of radiation dose led to decreased late resistance (*p* < 0.01) at both the 5 and 20 Gy doses, signaling greater cell death. U87 cells, conversely, showed no statistically significant changes to late resistance following treatment.

Though ECIS yields robust data, its measurements represent the aggregate of many cellular effects, including physical, morphological, and behavioral effects. Therefore, effects on cell death were more directly measured using clonogenic assays, which isolated cell survival data more effectively and over a longer period than ECIS alone.

### 3.2. Radiation with Concurrent TMZ and Durvalumab Does Not Significantly Affect T98G Cell Survival Relative to Radiation Only

**Figure 8:**
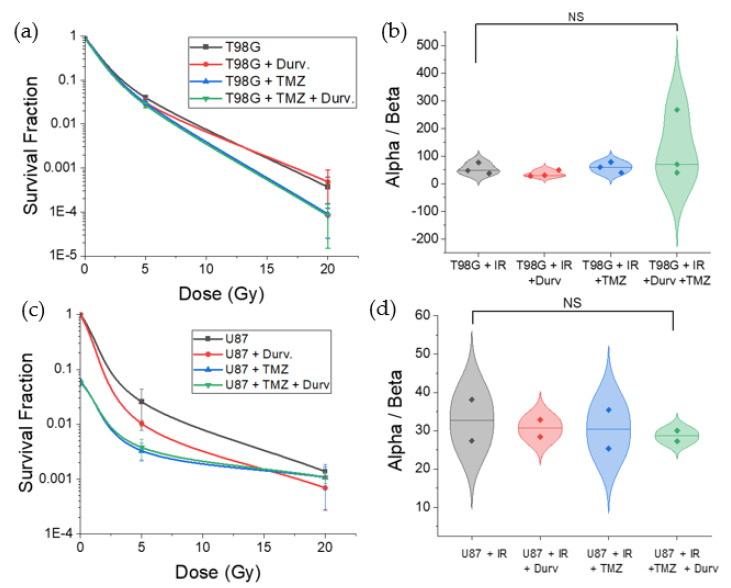
Survival curve and calculated alpha/beta ratio. (**a**) Survival curve for T98G cells treated with radiation at 0, 5, and 20 Gy alone, with concurrent temozolomide, durvalumab, and both. (**b**) Calculated alpha/beta ratio for (**a**). (**c**) Survival curve for U87G cells treated with radiation at 0, 5, and 20 Gy alone, with concurrent temozolomide, durvalumab, and both. (**d**) Calculated alpha/beta ratio for (**c**). Overall, increased radiation dose led to greater cell death (*p* < 0.001) for both cell lines. The addition of each agent to radiation showed no significant change to cell survival relative to the radiation-only conditions for T98G, but for U87, the addition of TMZ decreased cell survival (*p* < 0.0001) at 0 Gy, and marginally, but not significantly, decreased cell survival at higher radiation doses. NS non-significant.

Clonogenic assays are the gold standard in the measurement of cell survival following treatment. When tested at 0, 5, and 20 Gy for each treated case, increased radiation dose decreased cell survival (*p* < 0.001), irrespective of other treatments used concurrently (Figure 8), for both T98G and U87. The survival fractions were 0.93 ± 0.03, 0.040 ± 0.003, and $3.7\times 10^{-4}\pm 2.5\times 10^{-4}$ at each dose level, respectively, for T98G, and 0.95 ± 0.04, 0.026 ± 0.018, and $1.3\times 10^{-3}\pm 3.0\times 10^{-4}$ at each dose level for U87 MG. This effect was dominant for all trials, regardless of the presence of TMZ, durvalumab, or both agents.

However, T98G treated with TMZ, durvalumab, and both agents concurrently resulted in no significant differences in cell survival at each radiation dose as compared to the radiation-only condition. Furthermore, α/β showed no significant changes with the addition of TMZ and durvalumab to radiation, suggesting little effect of these agents as radiosensitizers for glioblastoma cells. However, the calculated *α*/*β* exceeded reasonable ratios for mammalian cells [56], suggesting that the present data may not have been accurately described by the linear-quadratic (LQ) model.

### 3.3. Radiation with Concurrent TMZ Decreases U87 MG Cell Survival

Results for U87 cells treated with radiation were similar to those for T98G cells (Figure 8). At the 5 and 20 Gy doses, cell survival dropped significantly (*p* < 0.0001). With the addition of durvalumab, there was no significant change to cell survival at any radiation dose, showing that durvalumab had no significant effect on cell survival for U87. When treated with TMZ, however, U87 cells had drastically decreased cell survival at 0 Gy for both TMZ and TMZ + durvalumab conditions (*p* < 0.0001). At the 5 Gy dose, the addition of TMZ marginally decreased cell survival, but the change was not statistically significant. At the 20 Gy dose level, the survival of cells treated with TMZ or TMZ and durvalumab was indistinguishable from the conditions not treated with TMZ. Therefore, for U87, TMZ is effective in boosting cell killing at low doses, but at increased radiation doses up to 20 Gy, treatment with radiation remains the dominant factor in cell killing.

The calculated *α*/*β* ratio for U87 MG showed similar results to T98G. The ratio was not significantly affected by the addition of either temozolomide, durvalumab, or both agents concurrently, showing little effect on radiosensitization for either agent. *α*/*β* averaged 30.6 ± 10.8 for U87, which still exceeded estimates of *α*/*β* for gliomas, estimated at around 10.0 [56]. However, within the error margins of our data, our results are within the expected order of magnitude, even though our survival data for U87 MG may not be best fitted using the LQ model.

## 4. Discussion

### 4.1. Towards Patient-Adaptive Therapy

In this work, we characterized the cellular-level effects of treatment with radiation, the immune-modulating agent temozolomide, and the immune checkpoint inhibitor durvalumab, for glioblastoma. Glioblastoma is extremely malignant, and, despite all efforts, there is no cure [57,58]. Researchers and physicians have long sought to improve therapeutic outcomes for glioblastoma, which currently has a median survival of around 15 months [3,7]. Following successes in the treatment of other cancers, including metastatic melanoma and non-small-cell lung cancer, immunotherapeutic agents acting as immune checkpoint inhibitors have been applied toward a variety of cancers, including glioblastoma [18,20,59]. One such agent, durvalumab, is currently undergoing phase I/II clinical trials for the treatment of glioblastoma;^2^ however, its effects on glioblastoma and healthy tissues in the brain have not yet been fully characterized at the cellular level. Building on previous findings that some chemotherapeutic agents and radiation may have unforeseen pro-metastatic effects [60,61], we applied our in vitro assays toward characterizing the cellular-level effects of durvalumab and temozolomide in radioimmunotherapy for glioblastoma. The nature of our multiparametric in vitro testing allowed us to monitor and quantify changes in various cellular characteristics, including cell death, motility, adhesion, and morphology, in real time following treatment, building a basis for patient-adaptive therapy.

### 4.2. Effect of Radioimmunotherapy on Cell Migration

Results showed that T98G cells treated with both radiation and temozolomide had increased *ROM* relative to untreated cells (Figure 2), mainly at 20 Gy, indicating that T98G cells migrated more following treatment than untreated cells (*p* < 0.01). These results suggest that radiation alone and radiation in combination with the immune-modulating agent, TMZ, may increase cell migration, in vivo. It has indeed been reported that subcurative radiation increases the invasion and migration of primary glioblastoma cells in vivo [62]. For GBM patients, this suggestion may account for local invasion despite treatment with TMZ and radiation. Thus, a patient with a tumor comprising cells similar to T98G may be at increased risk of tumor recurrence following treatment, should cells survive therapy and subsequent immune responses. U87 cells treated with radiation and temozolomide showed different trends in *ROM* as compared to treated T98G. Microsphere formation by U87 cells in addition to migration make the interpretation of our results less straightforward and also present an opportunity for considering well-known in vivo tumor heterogeneity [63] even in our in vitro assays. Interestingly, durvalumab does not significantly affect T98G or U87 cell migration, signaling that it may not worsen GBM invasiveness.

### 4.3. Effect of Radioimmunotherapy on Cell–Cell Adhesion

The disc model of ECIS by Giaever and Keese [46,50,55] includes the barrier function *R_b_*, which describes the presence and function of tight junctions (TJ) between cells in an adherent monolayer. Tighter cell junctions yield a higher reading of *R_b_*, whereas compromised tight junctions yield a lower *R_b_* [55]. TJ function has several impacts on metastasis. First, in the metastatic cascade, a primordial step is the loss of cell–cell adherence. A loss of adhesion signals compromised TJs. Once cells detach from the primary tumor, metastatic cells invade nearby tissues and enter and exit the circulatory system through intravasation and extravasation, respectively, which involves cells slipping through gaps between healthy cells. Therefore, though only readily applicable to monolayer-forming cells, analysis of the barrier functions in heathy and cancerous cells alike may reveal metastatic potential involving cell shedding from primary tumors and movement through healthy tissues. Irradiated T98G at the 5 and 20 Gy doses had decreased *R_b_* relative to untreated cells (*p* < 0.05). This result suggests that radiation leads to compromised tight junctions, and therefore may have other pro-metastatic effects via cell shedding and invasion, in addition to spurring migration. Concurrent TMZ further decreased the barrier function at the 5 Gy dose (*p* < 0.05) and did not significantly alter *R_b_* at the 20 Gy dose level. These results suggest that concurrent radiation and TMZ may augment pro-metastatic effects involving cell shedding and local invasion relative to radiation alone at low doses. Because glioblastoma cells in patients tend to invade nearby tissues naturally, there may be significant cell populations in nearby tissues which reside in the periphery of treated radiation fields in patients, and therefore receive sublethal doses of radiation. For these cells, the pro-metastatic effects of treatment may spur tumor recurrence. Since concurrent durvalumab led to marginal increases in the measured *R_b_*, including a statistically significant increase (*p* < 0.05) at the 5 Gy dose level compared to radiation alone, radioimmunotherapy with durvalumab may be part of the solution to local invasion and tumor recurrence. Durvalumab may thus hold potential as a soft antimetastatic therapy in addition to its function as an immune checkpoint inhibitor for glioblastoma.

### 4.4. Effect of Radioimmunotherapy on Cell Survival

We probed cell survival in real time and analyzed cells at 14-day and 21-day periods. Concurrent radiation treatment with TMZ and TMZ + durvalumab led to marginally lower, but not statistically significant, changes in cell survival relative to irradiated conditions for T98G, but significantly decreased cell survival for U87 (*p* < 0.0001) at 0 Gy. Additionally, at a low radiation dose (5 Gy), we observed a significant decrease in cell survival for U87 MG treated with TMZ (*p* < 0.0001), which was not observed with T98G, showcasing the differing efficacy of treatment against the same cancer type (glioblastoma). This result agrees with historical data which credit TMZ with only marginally increasing median survival for glioblastoma patients [64,65,66,67]. These findings therefore support a need for patient-specific targeted therapy, where efficacy can be evaluated prior to commitment to a treatment regimen.

### 4.5. Limitations and Outlook

For ECIS experiments and analysis, not all cells, including those taken from patients, may form complete monolayers. Thus, adaptations and better modeling are needed to enable the quantification of cell characteristics for a wider variety of cell morphologies and origins. Furthermore, the tumor microenvironment is widely known to affect cell behavior. Although our in vitro studies allowed for the isolation of variables and simplified visualizations of processes due to the direct impact of therapeutic agents on the cancer cells, processes which would otherwise escape some in vivo observations, we have not yet included immune-system-level interactions. Future work will have to incorporate co-cultures with immune cells and in three-dimensional culture environments. Other PD-1 immune checkpoint inhibitors, such as nivolumab and pembrolizumab, feature in several phase I clinical trials in radioimmunotherapy for glioblastoma and would therefore benefit from similar in vitro testing done in this work, for the rapid supplementary evaluation of RIT against brain tumors [2]. This work provides a framework for the advancement of radioimmunotherapy for brain tumors using in vitro assays. Care providers may test agents and therapies against patients’ cancers prior to treatment, which may improve therapeutic outcomes by helping to determine optimal strategies.

## 5. Conclusions

We have used in vitro assays to detect changes to cell behavior and cell death for both standard-of-care therapy (TMZ) against glioblastoma, and radioimmunotherapy with durvalumab, which is undergoing phase I/II clinical trials. Durvalumab did not alter cell migration as both radiation and TMZ did for the two glioblastoma cell lines (T98G and U87) tested. Cell–cell adhesion increased (*p* < 0.05) with durvalumab at a low radiation dose (5 Gy), unlike TMZ and radiation, which lowered cell–cell adhesion, in T98G cells. Thus, durvalumab shows a potential antimetastatic or anti-invasion effect at sublethal radiation doses. Though radiation had the most significant reduction in cell survival (*p* < 0.0001) in our testing, in vitro assays that include the immune system in co-cultures will better reveal the cancer cell killing and tumor control abilities of the immune-modulating and immunotherapeutic agents used in this work, as well as similar agents under clinical trials. The methods developed here may provide a framework for patient-specific targeted cancer therapy. We envisage our in vitro assays to be applicable to cells from patient biopsies, to evaluate the potential efficacy of treatment strategies for the overall improvement of treatment outcomes against aggressive cancers such as glioblastomas.

## Data Availability

Data is contained within the article and within supplementary material.

## Supplementary Materials

The following supporting information can be downloaded at: https://www.mdpi.com/article/10.3390/biomedicines10081796/s1, Figure S1: Adherent cell culture procedure; Figure S2: Images of the Faxitron CellRad X-ray machine; Figure S3: The Electric Cell Impedance Sensor (ECIS); Figure S4: ECIS 8W10E+ array and T98G cells growing to cover the bottom of the cell array; Figure S5: Loading and preparation of the ECIS; Figure S6: Late barrier function for the first two trials (N1, N2) of T98G + IR + durvalumab.
