# Supplementary material for: Development of In Vitro Assays for Advancing Radioimmunotherapy against Brain Tumors
Source: Biomedicines. 2022 Jul 26;10(8):1796. doi: 10.3390/biomedicines10081796 (PMC9394411; doi:10.3390/biomedicines10081796)
Supplement: Supplementary file 1 [file biomedicines-10-01796-s001.zip › biomedicines-1737882-supplementary.pdf]

biomedicines

Supplementary Information

Development of *in vitro* assays for advancing radioimmunotherapy against brain tumors

Yohan Walter[1], Anne Hubbard[1], Allie Benoit[1], Erika Jank[1], Olivia Salas[1], Destiny Jordan[2] and Andrew Ekpenyong[1,*]

1 Department of Physics, Creighton University, Omaha, USA; yohanwalter@creighton.edu (YW); annehubbard1@creighton.edu (AH); alliebenoit@creighton.edu (AB); erikajank@creighton.edu (EJ); oliviasalas@creighton.edu (OS); andrewekpenyong@creighton.edu (AE)
2 Department of Biology, Creighton University, Omaha, USA; destinyjordan@creighton.edu (DJ).
* Correspondence: andrewekpenyong@creighton.edu; Tel. 402-280-2208)

Citation: Walter, Y.; Hubbard, A.; Benoit, A.; Jank, E.; Salas, O.; Jordan, D.; Ekpenyong, A. Development of In Vitro Assays for Advancing Radioimmunotherapy against Brain Tumors. *Biomedicines* **2022**, *10*, 1796. https://doi.org/10.3390/biomedicines10081796

Academic Editors: Eric J. Lehrer and Daniel M. Trifiletti

Received: 6 May 2022
Accepted: 21 July 2022
Published: 26 July 2022

Publisher's Note: MDPI stays neutral with regard to jurisdictional claims in published maps and institutional affiliations.

Copyright: © 2022 by the authors. Licensee MDPI, Basel, Switzerland. This article is an open access article distributed under the terms and conditions of the Creative Commons Attribution (CC BY) license (https://creativecommons.org/licenses/by/4.0/).

Supplementary Figures

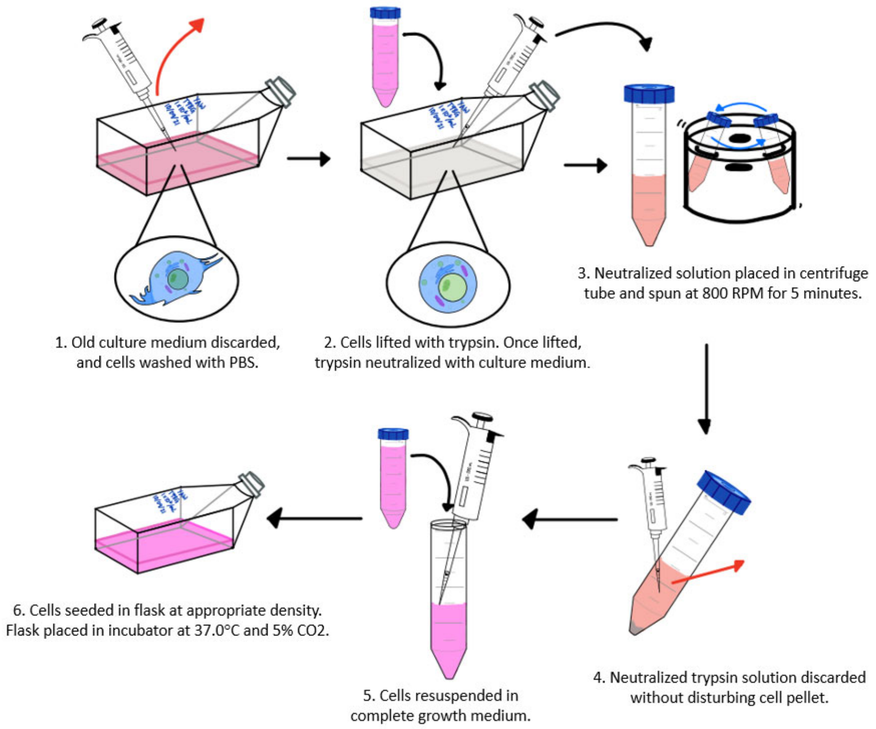

Figure S1. Adherent cell culture procedure. After discarding old medium and rinsing with phosphate buffered saline (PBS), cells were chemically lifted with trypsin. Following lifting, trypsin was neutralized with culture medium and the solution was placed in a centrifuge spun at 800 RPM for 5 minutes. Simultaneously, cells were counted using a hemacytometer or automatic cell counter. The neutralized trypsin solution was then extracted without disturbing the cell pellet, which was later resuspended in complete growth medium and aliquoted into a new culture flask at appropriate cell density.

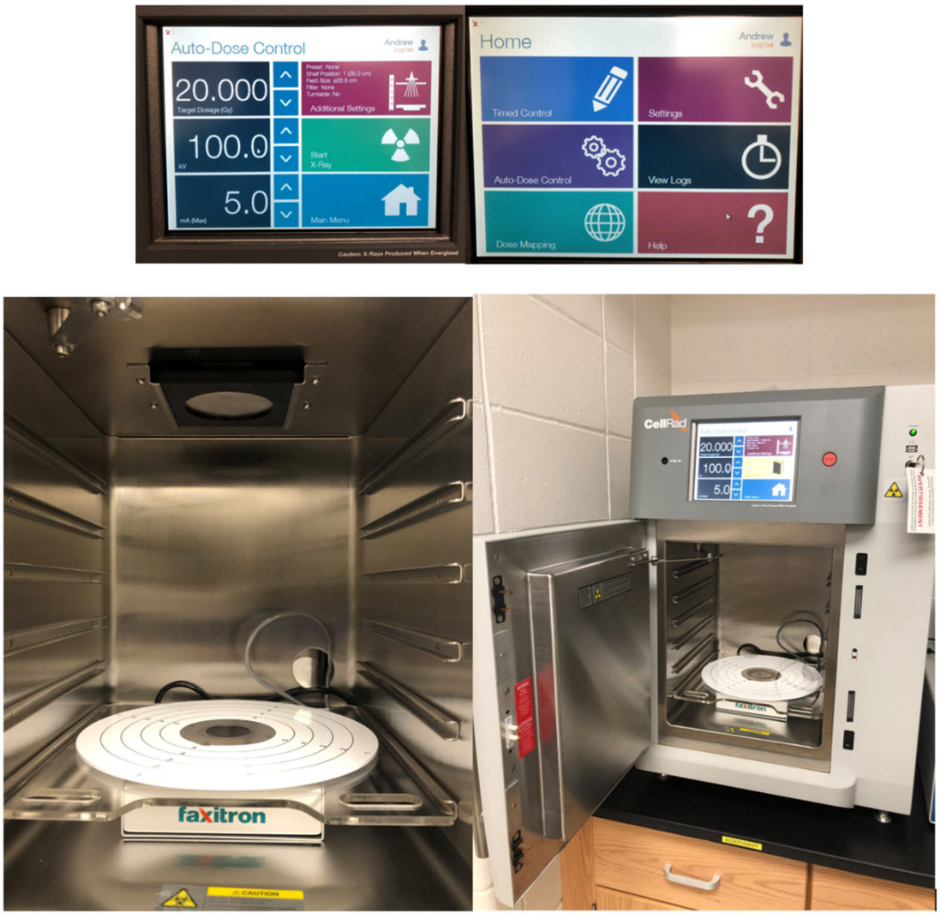

Figure S2. Images of the Faxitron CellRad x-ray machine. Top left: Auto-Dose Control interface and settings. The user can toggle settings in amount of accumulated radiation dose, kVp, and tube current. Top right: Home screen of CellRad interface. Bottom left: Interior of the machine. Specimen is placed in the center of the white turntable. Beam exits the source through the aperture on the ceiling of the machine. Bottom right: Full image of the Faxitron CellRad.

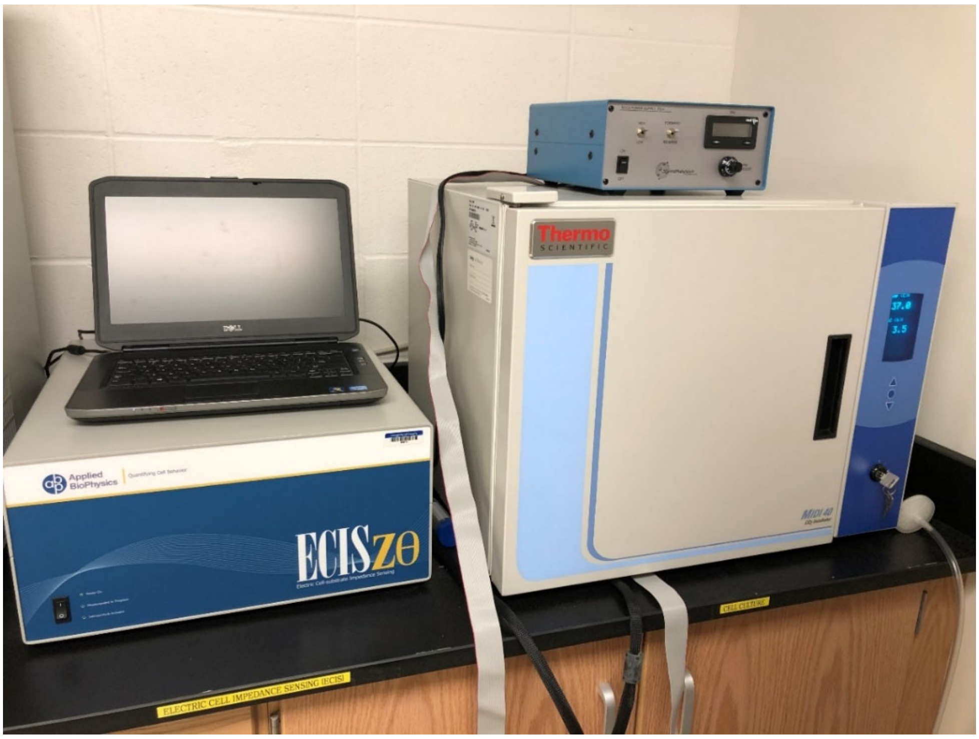

Figure S3. The Electric Cell Impedance Sensor (ECIS). We used the Z-theta system (Applied BioPhysics, New York, USA) shown here. Array station is placed inside the incubator (right).

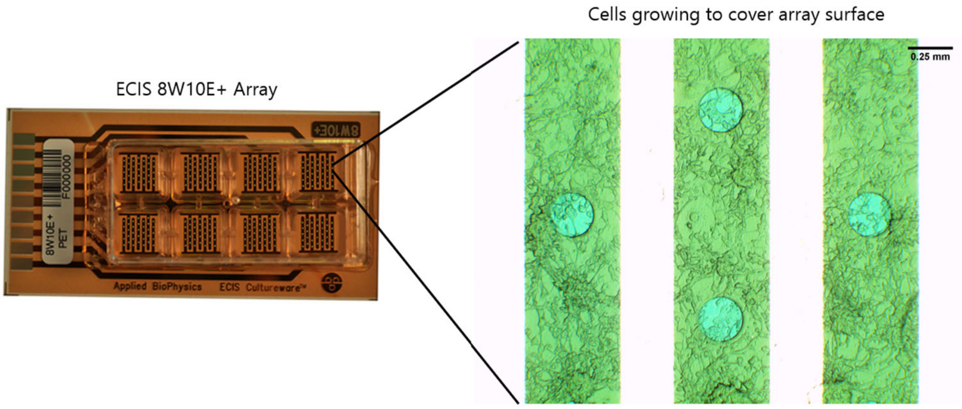

Figure S4. ECIS 8W10E+ array and T98G cells growing to cover the bottom of the cell array. Cell coverage directly affects measured impedance by increasing resistive flow with increasing coverage.

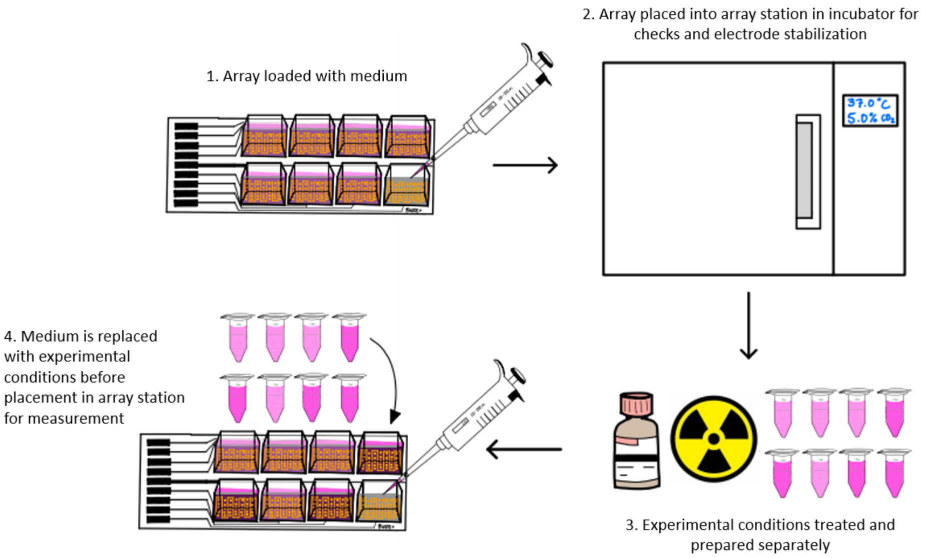

Figure S5. Loading and preparation of the ECIS. 1. 8W10E+ array is loaded with medium as a substrate for built-in "check" and "stabilize" functions prior to experiment. 2. Array is loaded into incubator-housed array station for calibrations. 3. Experimental conditions are prepared separately as needed. 4. Array is removed from incubator station and medium is replaced by experimental conditions. Array is then placed back into the station and allowed to stabilize for 15 minutes before a final "check" and measurement start.

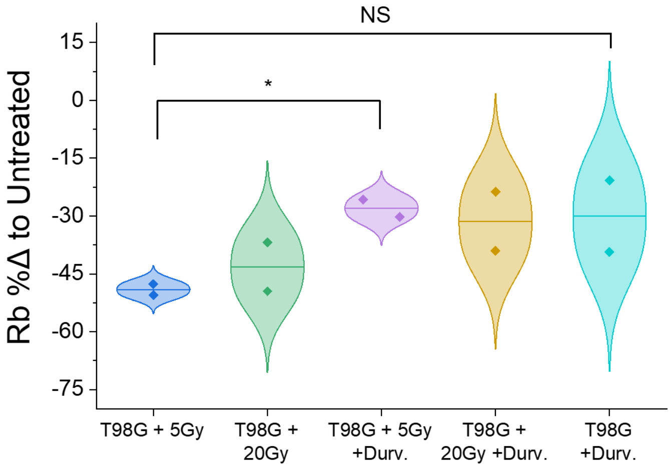

Figure S6. Late barrier function for the first two trials (N1, N2) of T98G + IR + durvalumab. Relative to the untreated condition, all 5 and 20 Gy-irradiated conditions showed decreased late barrier function ($p < 0.05$), signaling compromised cell-cell adhesion.
